# Influence of the Bias Voltage on Effective Electron Velocity in AlGaN/GaN High Electron Mobility Transistors

**DOI:** 10.3390/mi15091148

**Published:** 2024-09-13

**Authors:** Guangyuan Jiang, Peng Cui, Chen Fu, Yuanjie Lv, Ming Yang, Qianding Cheng, Yang Liu, Guangyuan Zhang

**Affiliations:** 1 School of Information Science and Electrical Engineering, Shandong Jiaotong University, Jinan 250357, China; 2 Institute of Novel Semiconductors, Shandong University, Jinan 250101, China; 3 National Key Laboratory of Application Specific Integrated Circuit (ASIC), Hebei Semiconductor Research Institute, Shijiazhuang 050051, China; 4 Beijing Orient Institute of Measurement and Testing, Beijing 100094, China

**Keywords:** AlGaN/GaN HEMTs, bias voltage, effective electron velocity, polarization coulomb field scattering

## Abstract

The small-signal S parameters of the fabricated double-finger gate AlGaN/GaN high electron mobility transistors (HEMTs) were measured at various direct current quiescent operating points (DCQOPs). Under active bias conditions, small-signal equivalent circuit (SSEC) parameters such as *R_s_* and *R_d_*, and intrinsic parameters were extracted. Utilizing *f*_T_ and the SSEC parameters, the effective electron velocity ($\nu_{e-eff}$) and intrinsic electron velocity ($\nu_{e-\mathrm{int}}$) corresponding to each gate bias (V_GS_) were obtained. Under active bias conditions, the influence mechanism of V_GS_ on $\nu_{e-eff}$ was systematically studied, and an expression was established that correlates $\nu_{e-eff}$, $\nu_{e-\mathrm{int}}$, and bias-dependent parasitic resistances. Through the analysis of the main scattering mechanisms in AlGaN/GaN HEMTs, it has been discovered that the impact of V_GS_ on $\nu_{e-eff}$ should be comprehensively analyzed from the aspects of $\nu_{e-\mathrm{int}}$ and parasitic resistances. On the one hand, changes in V_GS_ influence the intensity of polar optical phonon (POP) scattering and polarization Coulomb field (PCF) scattering, which lead to changes in $\nu_{e-\mathrm{int}}$ dependent on V_GS_. The trend of $\nu_{e-\mathrm{int}}$ with changes in V_GS_ plays a dominant role in determining the trend of $\nu_{e-eff}$ with changes in V_GS_. On the other hand, both POP scattering and PCF scattering affect $\nu_{e-eff}$ through their impact on parasitic resistance. Since there is a difference in the additional scattering potential corresponding to the additional polarization charges (*APC*) between the gate-source/drain regions and the region under the gate, the mutual effects of PCF scattering on the under-gate electron system and the gate-source/drain electron system should be considered when adjusting the PCF scattering intensity through device structure optimization to improve linearity. This study contributes to a new understanding of the electron transport mechanisms in AlGaN/GaN HEMTs and provides a novel theoretical basis for improving device performance.

## 1. Introduction

Gallium nitride (GaN) materials are typical wide-bandgap semiconductor materials [1]. AlGaN/GaN HEMTs based on GaN materials are outstanding representatives of the new generation of semiconductor devices [2,3,4]. Owing to their superior performance, such as high electron velocity and high critical breakdown electric field, they hold broad market application prospects in high-frequency and high-power fields, including aerospace and mobile communication, among others [5,6,7]. Despite a series of scientific and technological breakthroughs in the study of AlGaN/GaN HEMTs, their power output and linearity have not yet fully reached the expected values due to non-ideal factors [8,9,10]. This has become an important factor restricting the large-scale commercial application of AlGaN/GaN HEMTs.

The channel electron velocity has a significant impact on device performance [11]. The electron velocity and maximum current-gain cutoff frequency (*f*_T_) of AlGaN/GaN HEMTs remain a controversial issue [9]; the rapid decrease in *g*_m_ and *f*_T_ with increasing gate bias is believed to be related to effective electron velocity ($\nu_{e-eff}$) [11,12]. However, much of the current research mainly focuses on the peak of electron velocity [9,13], which cannot fully reflect the operating mechanism of the device. There is relatively little research on the bias dependence of electron velocity. In the limited number of studies currently available on the bias dependence of electron velocity, the extraction of bias-related electron velocities is based on small-signal model parameters obtained through the COLD-FET method, without considering the bias dependence of parasitic resistances [14]. Due to the influence of bias voltage on the two-dimensional electron gas (2DEG) of the access area, the parasitic source and drain resistances (*R_s_* and *R_d_*) of AlGaN/GaN HEMTs have bias dependence [15,16,17]. *R_s_* and *R_d_* are important reasons for the inconsistency between the external effective parameters and intrinsic parameters of the device. Therefore, when studying the effect of bias voltage on $\nu_{e-eff}$, it is necessary to consider the bias dependence of *R_s_* and *R_d_*. Polar optical phonon (POP) scattering and polarization Coulomb field (PCF) scattering are the most important scattering mechanisms for AlGaN/GaN HEMTs, and their intensity is affected by bias voltage [18,19,20]. Therefore, when studying the effect of bias voltage on $\nu_{e-eff}$ in AlGaN/GaN HEMTs, it is necessary to systematically analyze the relationship between gate bias, parasitic resistance, scattering mechanism, and $\nu_{e-eff}$.

In this study, double-finger gate AlGaN/GaN HEMTs suitable for high-frequency applications were fabricated, and the broadband S parameters were measured under different gate bias conditions. Small-signal equivalent circuit (SSEC) parameters such as *R_s_* and *R_d_*, and intrinsic parameters were extracted under active bias conditions. The intrinsic electron velocity ($\nu_{e-\mathrm{int}}$) dependent on gate bias is calculated using these SSEC parameters. Moreover, the $\nu_{e-eff}$ corresponding to each gate bias voltage is obtained through the *f*_T_. We analyzed the mechanism by which bias voltage affects $\nu_{e-eff}$ and established a correlation expression between $\nu_{e-eff}$ and $\nu_{e-\mathrm{int}}$. This study is beneficial for understanding the electron transport mechanism of AlGaN/GaN HEMTs from a new perspective and provides a new theoretical basis for improving device performance, such as linearity.

## 2. Experiments

AlGaN/GaN heterostructure wafers were grown on 4H-SiC substrates via MOCVD. Above the substrate are a 1000 nm GaN buffer layer, 400 nm undoped GaN, 0.8 nm AlN, 21 nm Al_0.26_Ga_0.74_N, and 3 nm GaN. The electron mobility and 2DEG density of the wafer, obtained by Hall measurement, are 2073 [cm^2^/(V·s)] and 1.09 × 10^13^ cm^−2^.

**Figure 1:**
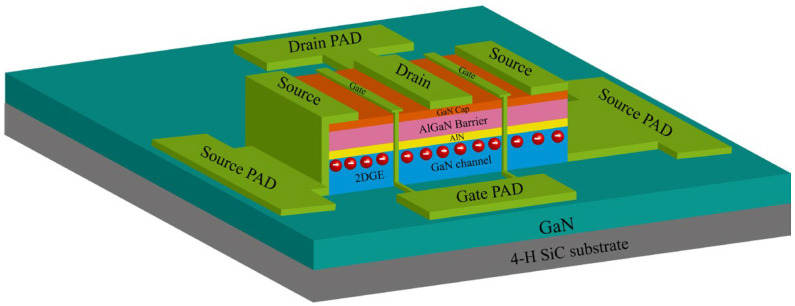
Schematic diagram of the AlGaN/GaN HEMTs used in this study.

The structure of the AlGaN/GaN HEMTs used in this study is shown in Figure 1. The source and drain of the device are Ohmic contacts, which are formed by stacking Ti/Al/Ni/Au on AlGaN/GaN heterostructure wafers and then rapidly annealing in an N_2_ environment. The gate is a Schottky contact, manufactured by depositing Ni/Au after electron beam lithography. The device is a central gate; the gate length (*L_G_*) is 300 nm, and a gate width (*W_G_*) is 40 × 2 μm. The device with 1 μm gate-source spacing (*L_GS_*) is named as Sample 1, and the device with 2 μm *L_GS_* is named as Sample 2. The I-V characteristics and S parameters were measured using the Keysight B1500A Semiconductor Device Parameter Analyzer(Keysight Technologies Inc., Santa Rosa, CA, USA) and the Keysight PNA-X Vector Network Analyzer.(Keysight Technologies Inc., Santa Rosa, CA, USA).

## 3. Results and Discussion

**Figure 2:**
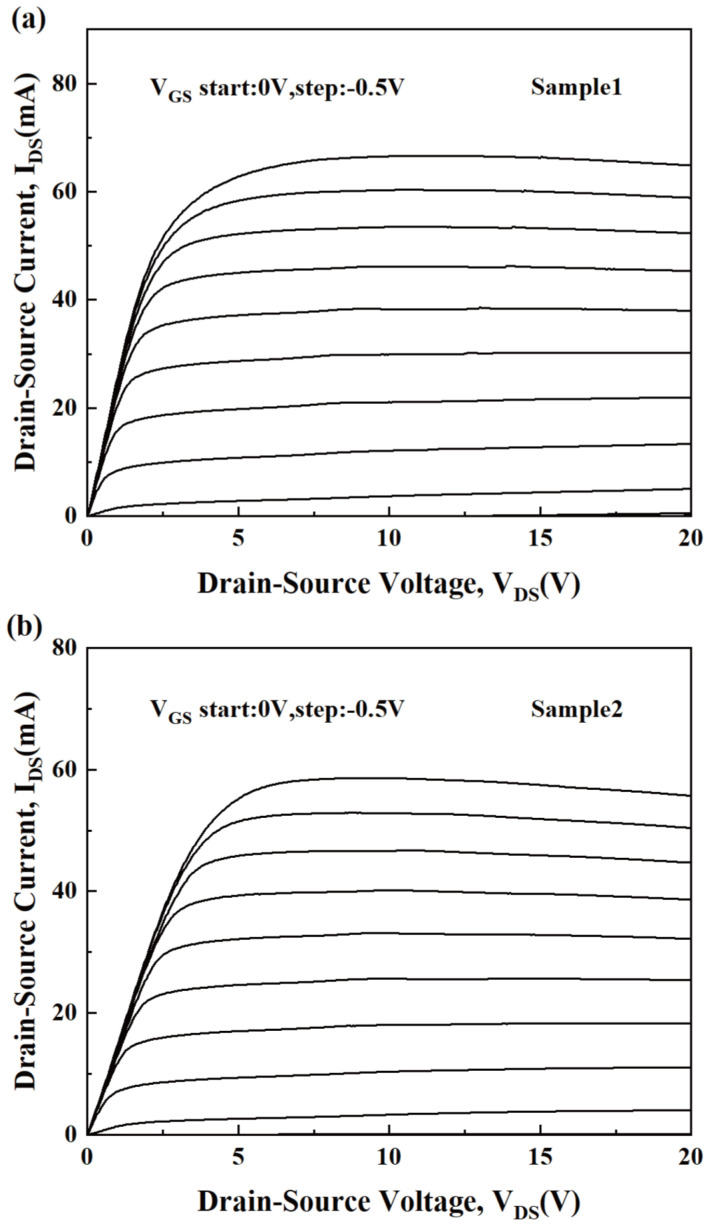
The measured I-V characteristics of (**a**) Sample 1 and (**b**) Sample 2.

**Figure 3:**
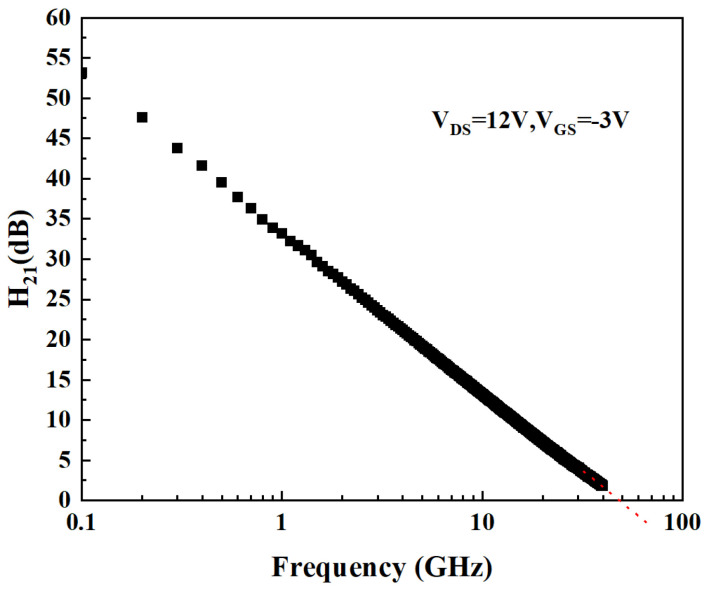
Method for obtaining *f*_T_ of AlGaN/GaN HEMTs through H_21_ (taking the *f*_T_ of Sample 1 at DCQOPs of V_DS_ = 12 V, V_GS_ = −3 V as an example; *f*_T_ = 48.6 GHz).

**Figure 4:**
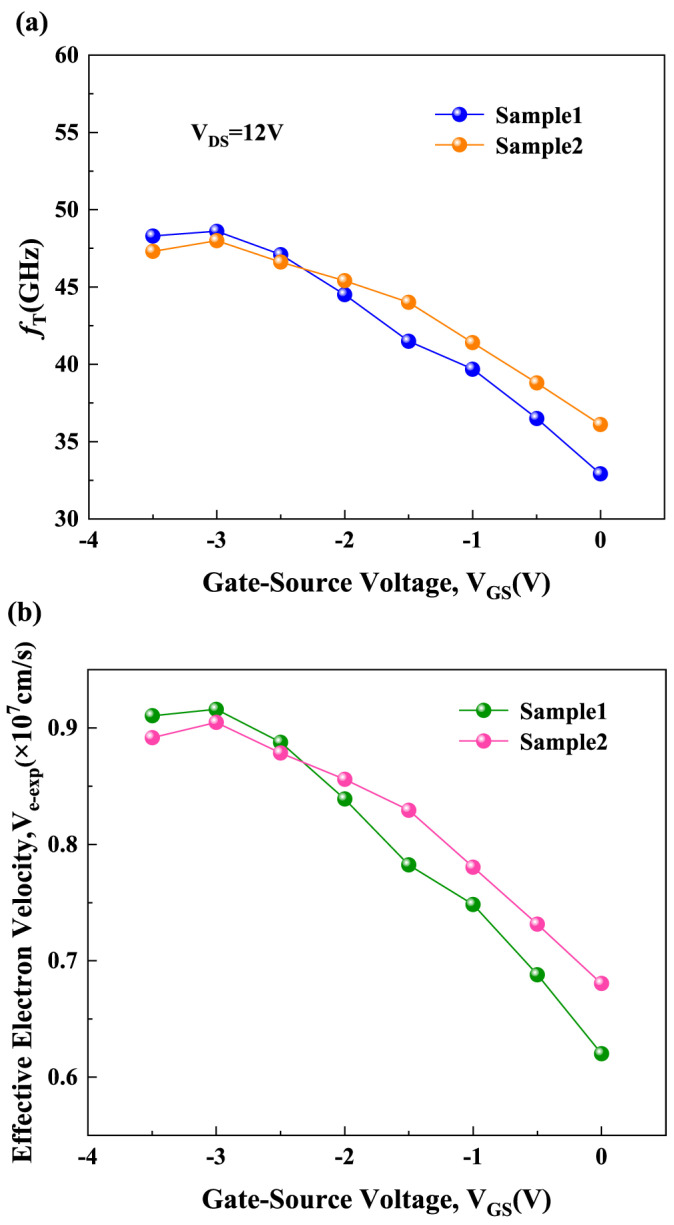
(**a**) The *f*_T_ corresponding to each gate bias and (**b**) the $\nu_{e-\exp}$ corresponding to each gate bias for Sample 1 and Sample 2.

The I-V characteristics of Sample 1 and Sample 2 are shown in Figure 2. The points with V_DS_ = 12 V and V_GS_ = 0 to −3.5 V (step: −0.5 V) were chosen as the direct current quiescent operating points (DCQOPs) to measure the small-signal S parameters corresponding to each gate bias condition. The frequency range for small-signal S parameter measurement is 0.5 to 40 GHz. The small-signal S parameter is converted to the H-parameter, and the current-gain modulus H_21_ (dB) is obtained. Therefore, as shown in Figure 3 and Figure 4a, the *f*_T_ can be obtained by extrapolating H_21_ (dB) [21,22,23,24]. For AlGaN/GaN HEMTs, the external effective electron velocity (experimental value) can be expressed as follows [25]:

(1) $$\nu_{e-\exp}=2\cdot \pi \cdot f_{T}\cdot L_{G}$$

So, as shown in Figure 4b, the $\nu_{e-\exp}$ corresponding to each gate bias for the two samples can be obtained. From Figure 4b, it can be seen that $\nu_{e-\exp}$ reaches its peak at a V_GS_ of −3 V, and gradually decreases as V_GS_ increases from −3 V to 0 V. The phenomenon of $\nu_{e-\exp}$ decreasing with increasing V_GS_ will seriously affect the linearity of the device.

The phenomenon of effective electron velocity decreasing with increasing V_GS_ is related to the intrinsic electron velocity ($\nu_{e-\mathrm{int}}$) and *R_s_* and *R_d_*, which are related to V_GS_. The $\nu_{e-\mathrm{int}}$ of AlGaN/GaN HEMTs can be expressed as follows [26]:

(2) $$\nu_{e-\mathrm{int}}=\frac{g_{m-\mathrm{int}}}{C_{gs}+C_{gd}}\cdot L_{G}$$

**Figure 5:**
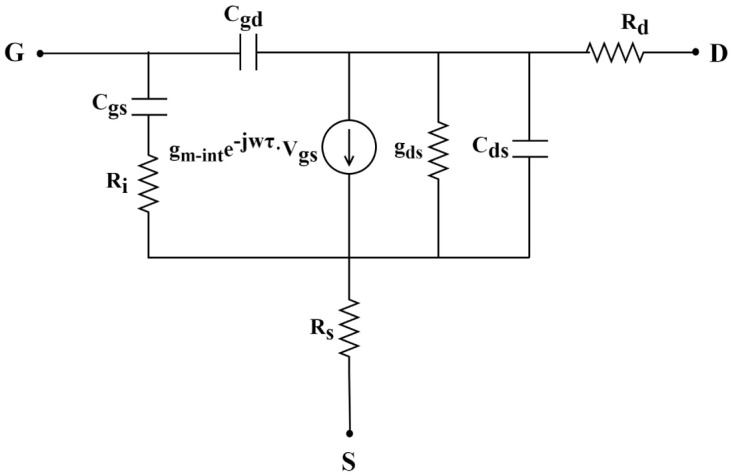
Topology diagram of SSEC for AlGaN/GaN HEMTs (parasitic parameters unrelated to bias are not depicted in this figure).

**Figure 6:**
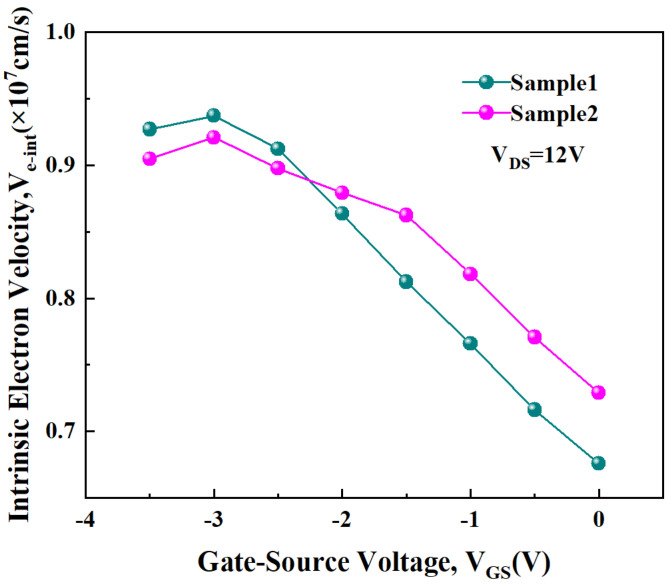
The $\nu_{e-\mathrm{int}}$ corresponding to each gate bias for Sample 1 and Sample 2.

Among them, $g_{m-\mathrm{int}}$ is the intrinsic transconductance, and $C_{gs}$ and $C_{gd}$ are intrinsic gate-source and gate-drain capacitors, which are directly extracted under active bias conditions based on the SSEC shown in Figure 5 [27,28,29,30,31]. Figure 6 shows the calculated $\nu_{e-\mathrm{int}}$ corresponding to each gate bias of Sample 1 and Sample 2.

From Figure 6, it can be seen that $\nu_{e-\mathrm{int}}$ reaches its peak at a V_GS_ of −3 V and then decreases significantly as V_GS_ increases from −3 V to 0 V. The magnitude of $v_{e-\mathrm{int}}$ is determined by the x-direction electric field under the gate (*E*_x_) and the scattering mechanisms. Existing research has shown that the change in the intensity of *E*_x_ is very slight when the gate bias is altered [32]. Therefore, the variation in $v_{e-\mathrm{int}}$ with V_GS_ is primarily determined by the scattering mechanisms. POP scattering and PCF scattering are the predominant scattering mechanisms in AlGaN/GaN HEMTs. As V_GS_ increases, both the temperature of polar optical phonons (T_POP_) and the density of the two-dimensional electron gas (*n*_2DEG_) increase, leading to enhanced POP scattering [33,34,35]. The enhancement of POP scattering intensity causes $v_{e-\mathrm{int}}$ to decrease with V_GS_. When V_GS_ < −3 V, both T_POP_ and *n*_2DEG_ are lower, resulting in weaker POP scattering, making PCF scattering the dominant mechanism. During the process of increasing V_GS_ from −3.5 V to −3 V, the inverse piezoelectric effect (IPE) weakens, leading to a reduction in the additional polarization charge (*APC*) and a decrease in PCF scattering, which results in an increase in $\nu_{e-\mathrm{int}}$. The variation in $\nu_{e-\mathrm{int}}$ with V_GS_ is an important factor influencing the variation in effective electron velocity with V_GS_. The above analysis indicates that the effects of POP scattering and PCF scattering on the variation trend of $\nu_{e-\mathrm{int}}$ with V_GS_ are opposite. Therefore, enhancing the PCF scattering strength corresponding to the electron system under the gate can reduce the magnitude of $\nu_{e-\mathrm{int}}$ at lower V_GS_ voltage ranges and compensate for the device linearity loss caused by the reduction in $\nu_{e-\mathrm{int}}$ due to the increased POP scattering caused by a higher V_GS_. This results in a more gradual change in $\nu_{e-\mathrm{int}}$ with V_GS_ and thereby improves the device’s linearity across the entire operating voltage range. Sample 2, with its larger *L_GS_* and *L_GD_* values, corresponds to a stronger additional scattering potential, which leads to more intense PCF scattering in the under-gate electron system. As a result, the variation in $\nu_{e-\mathrm{int}}$ with V_GS_ is more gradual, as illustrated in Figure 6.

**Figure 7:**
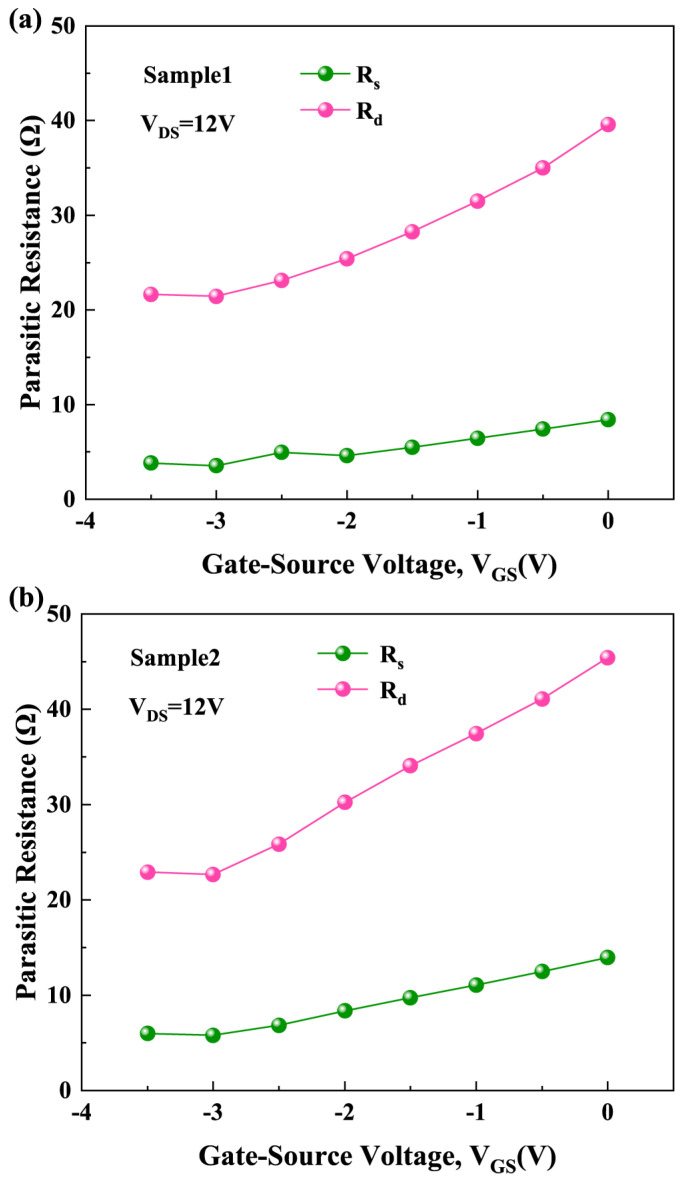
The *R_s_* and *R_d_* corresponding to each gate bias for (**a**) Sample 1 and (**b**) Sample 2.

**Figure 8:**
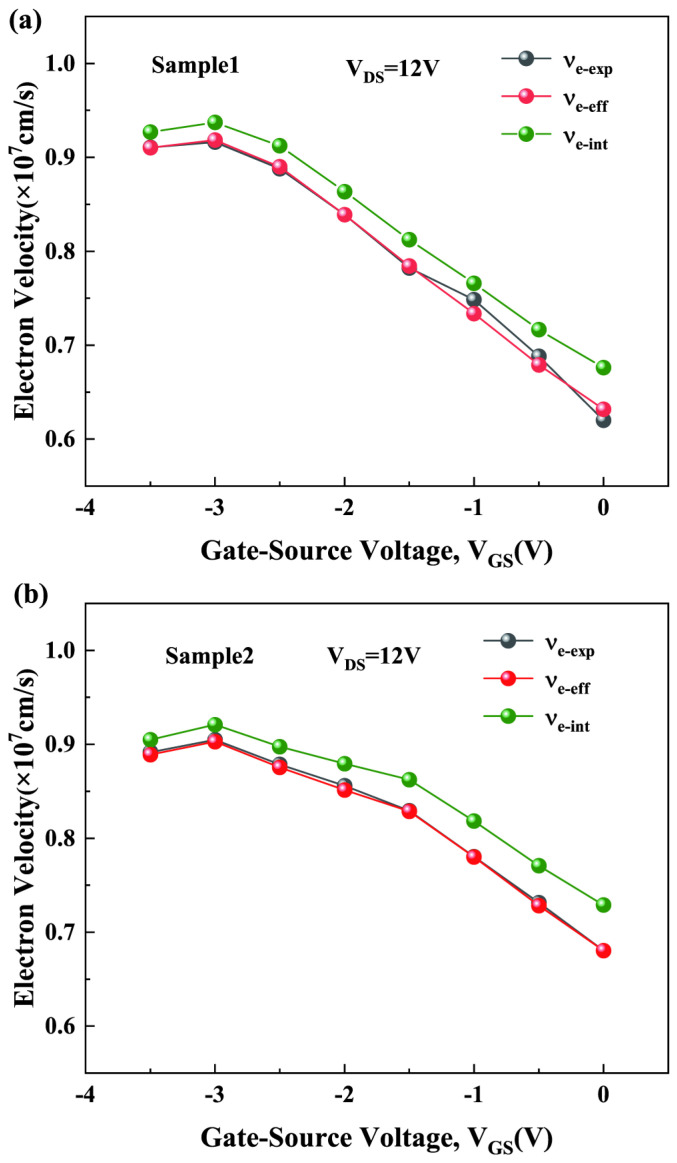
Comparison of $\nu_{e-eff}$ obtained through Formula (3) with $\nu_{e-\exp}$ from experiments and comparison of effective electron velocity with $\nu_{e-\mathrm{int}}$ for (**a**) Sample 1 and (**b**) Sample 2.

Figure 7 shows the *R_s_* and *R_d_* corresponding to each gate bias for Sample 1 and Sample 2. These values are directly extracted under active bias conditions based on the SSEC shown in Figure 5 [27,28,29,30,31]. Due to the modulation of *R_s_* and *R_d_* on the gate-source voltage and drain-source voltage [26,36], $\nu_{e-eff}$, the externally measured effective electron velocity, is less than $\nu_{e-\mathrm{int}}$. Considering these modulation effects, the relationship between $\nu_{e-eff}$, $\nu_{e-\mathrm{int}}$, and parasitic resistance can be expressed as follows:

(3) $$\nu_{e-eff}=\frac{\nu_{e-\mathrm{int}}}{\left[1+(\frac{\varepsilon_{0}\varepsilon_{AlGaN}W}{d})\cdot R_{s}\cdot \nu_{e-\mathrm{int}}\right]}-(R_{s}+R_{d})\cdot g_{ds}\cdot \nu_{e-\mathrm{int}}$$

where $\varepsilon_{0}$ is the dielectric constant of a vacuum, $\varepsilon_{AlG\mathrm{aN}}$ is the dielectric constant of AlGaN, *W* is the gate width, *d* is the barrier layer thickness, and *g_ds_* is the drain conductance. Figure 8 displays the $\nu_{e-eff}$ calculated using Formula (3) and the effective electron velocity obtained experimentally (denoted as $\nu_{e-\exp}$), illustrating that the two values are consistent.

**Figure 9:**
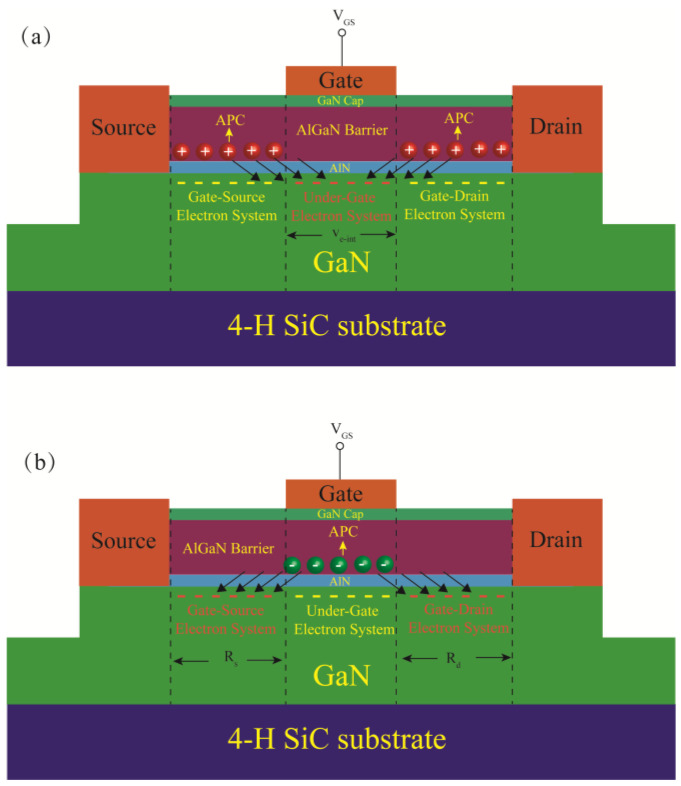
Schematic of the influence of the *APC* on the (**a**) under-gate electron system and (**b**) gate-source/drain electron system in the PCF scattering model.

Analysis of the relationship between the $\nu_{e-eff}$, parasitic resistances and $\nu_{e-\mathrm{int}}$ has revealed that both POP and PCF scattering can influence $\nu_{e-eff}$ by altering $\nu_{e-\mathrm{int}}$ and parasitic resistances. When the V_GS_ changes, the mechanisms by which POP and PCF scattering impact the $\nu_{e-\mathrm{int}}$ are similar to their effects on parasitic resistances [17,18]. As V_GS_ increases, the intensities of PCF and POP scattering exhibit opposite trends. Consequently, their counteracting effects can be utilized to moderate the changes in $\nu_{e-\mathrm{int}}$ and parasitic resistances induced by V_GS_, thus enhancing linearity during the entire operating voltage range. However, in the PCF scattering model, the drain-source channel is divided into two systems: the under-gate electron system and the gate-source/drain electron system [37]. As shown in Figure 9a, the impact of PCF scattering on $\nu_{e-\mathrm{int}}$ is realized by the scattering action of the *APC* present in the gate-source/drain regions on the electrons located in the area under the gate. The additional scattering potential generated by the *APC* present in the gate-source/drain regions can be expressed as follows [37]:

(4) $$\begin{array}{l} V_{APC-present\;in\;GS/GD}\left(x,y,z\right)=-\frac{e}{4\pi \varepsilon_{s}\varepsilon_{0}}\int_{-L_{\mathrm{GS}}-\frac{L_{G}}{2}}^{-\frac{L_{G}}{2}}dx^{\prime }\int_{0}^{W_{G}}\frac{\Delta \rho_{APC-GS}}{\sqrt{{\left(x-x^{\prime }\right)}^{2}+{\left(y-y^{\prime }\right)}^{2}+z^{2}}}dy^{\prime }\\ \;-\frac{e}{4\pi \varepsilon_{s}\varepsilon_{0}}\int_{\frac{L_{G}}{2}}^{L_{\mathrm{GD}}+\frac{L_{G}}{2}}dx^{\prime }\int_{0}^{W_{G}}\frac{\Delta \rho_{APC-GD}}{\sqrt{{\left(x-x^{\prime }\right)}^{2}+{\left(y-y^{\prime }\right)}^{2}+z^{2}}}dy^{\prime } \end{array}$$

where $\Delta \rho_{APC-GS}$ and $\Delta \rho_{APC-GD}$ are the amounts of *APC* present in the gate-source/drain regions. The $V_{APC-present\;in\;GS/GD}$ scatters the electrons located in the area under the gate, thereby affecting $\nu_{e-\mathrm{int}}$. Conversely, as shown in Figure 9b, the impact of PCF scattering on *R_s_* and *R_d_* is achieved through the scattering action of the *APC* present in the region under the gate on the electrons located in the gate-source/drain regions. The additional scattering potential generated by the *APC* present in the region under the gate can be expressed as follows [37]:

(5) $$V_{APC-present\;in\;G}\left(x,y,z\right)=-\frac{e}{4\pi \varepsilon_{s}\varepsilon_{0}}\int_{-\frac{L_{G}}{2}}^{\frac{L_{G}}{2}}dx^{\prime }\int_{0}^{W_{G}}\frac{\Delta \rho_{APC-G}}{\sqrt{{\left(x-x^{\prime }\right)}^{2}+{\left(y-y^{\prime }\right)}^{2}+z^{2}}}dy^{\prime }\;$$

where $\Delta \rho_{APC-G}$ is the amount of *APC* present in the region under the gate. The $V_{APC-present\;in\;G}$ scatters the electrons located in the gate-source/drain regions, thereby affecting *R_s_* and *R_d_*. When the under-gate electron system experiences strong PCF scattering, the PCF scattering in the gate-source/drain electron system might be weak. Therefore, when adjusting the intensity of PCF scattering to influence the device linearity by optimizing the device structure, the mutual effects of PCF scattering on the under-gate electron system and the gate-source/drain electron system should be considered. For the two samples in this study, since *L_GS_* is greater than *L_G_*, the under-gate electron system experiences stronger PCF scattering. Consequently, the impact of PCF scattering on $\nu_{e-\mathrm{int}}$ is greater than its impact on *R_s_* and *R_d_*.

## 4. Conclusions

In summary, based on the measured wideband small-signal S parameters of AlGaN/GaN HEMTs, the $\nu_{e-eff}$ is calculated using the *f*_T_ obtained. SSEC parameters such as *R_s_* and *R_d_* and intrinsic parameters were extracted under active bias conditions. And the $\nu_{e-\mathrm{int}}$ corresponding to each V_GS_ was also calculated. We analyzed the mechanism by which V_GS_ affects $\nu_{e-eff}$ and established an expression for the relationship between $\nu_{e-eff}$, $\nu_{e-\mathrm{int}}$, and parasitic resistances. By analyzing the main scattering mechanisms in AlGaN/GaN HEMTs, it was found that the impact mechanism of V_GS_ on $\nu_{e-eff}$ needs to be comprehensively analyzed from two aspects: $\nu_{e-\mathrm{int}}$ and parasitic resistances. On the one hand, the change in V_GS_ will affect the intensity of POP scattering and PCF scattering, leading to a change in $\nu_{e-\mathrm{int}}$. The trend of $\nu_{e-\mathrm{int}}$ changing with V_GS_ has a direct impact on $\nu_{e-eff}$ and plays a dominant role in the trend of $\nu_{e-eff}$ changing with V_GS_. On the other hand, due to the presence of parasitic resistance, $\nu_{e-eff}$ is smaller than $\nu_{e-\mathrm{int}}$. Due to the differences in *APC* between the gate-source/drain regions and the under-gate region, when optimizing the device structure to adjust the intensity of PCF scattering to influence device linearity, the mutual effects of PCF scattering on the under-gate electron system and the gate-source/drain electron system must be considered. This study comprehensively elucidates the impact mechanism of gate bias on $\nu_{e-eff}$ from both intrinsic and parasitic aspects, which is beneficial for understanding the electron transfer mechanism of AlGaN/GaN HEMTs from a new perspective and provides a new theoretical basis for improving the linear performance of the devices.

## Data Availability

The data presented in this work are available within the article.
